# A fibular notch approach for the treatment of ankle fractures involving the distal tibial plafond

**DOI:** 10.1186/s13018-021-02270-3

**Published:** 2021-02-08

**Authors:** Tong Liu, Yiheng Cheng, Wenqing Qu

**Affiliations:** Department of Orthopaedics, Yantaishan Hospital, NO.91 Jiefang Road, Zhifu District, Yantai, 264001 Shandong Province China

**Keywords:** Ankle fractures, Pilon fractures, Distal tibial plafond fractures, Surgical approach, Fibular notch

## Abstract

**Background:**

Although efficacy is related to many factors, the surgical approach is one of the most important intervention factors for complex ankle fractures. Ankle fractures involving the distal tibial plafond frequently present a surgical challenge in choosing which incisions will be best for surgical treatment. Here, we present an innovative fibular notch approach for the treatment of some specific ankle fractures and present a series of patients with either functional or radiographic outcomes.

**Methods:**

Twenty-two patients with distal tibial plafond fractures with concomitant fibular and distal tibiofibular syndesmosis injuries were treated through a fibular notch approach in this retrospective study. The details of the surgical technique were reviewed from the operative notes. Relevant data were reviewed from the medical records. The quality of fractures and syndesmosis reduction was examined using CT scans, and lateral stability of the ankle was assessed by physical examination and stress radiographs. The American Orthopedic Foot and Ankle Society Ankle-Hindfoot Scale (AOFAS) score was implemented for clinical functional assessment.

**Results:**

All surgeries were successfully performed via the fibular notch approach as the primary approach with excellent intraoperative visualization. Postoperative radiography revealed satisfying restoration of all fractures and syndesmosis. All fractures healed with an average time of 17.3 ± 3.6 weeks. Mild posttraumatic osteoarthritis (PTOA) was present in 4 patients. The average AOFAS score was 88.8 at the last follow-up.

**Conclusions:**

The fibular notch approach is a safe and reliable approach for the treatment of specific ankle fractures involving the distal tibial plafond. This approach provides excellent direct visualization of the fragments and articular surface without significantly increasing iatrogenic injuries. Satisfactory radiographic and clinical results were observed, and further clinical and anatomical studies are recommended to ascertain the feasibility of this approach in the treatment of complex distal tibial fractures.

## Background

Ankle fractures involving the distal tibial plafond, including traditional pilon fractures and atypical ankle fractures, are usually the result of combined axial load and shear force. Anatomical reduction of articular fragments and restoration of normal alignment as well as ankle stability have been considered to be the keys to success [1–4]. Orthopedic surgeons have been striving for ideal results; however, posttraumatic osteoarthritis (PTOA) and soft tissue-related complications have been bothersome to patients and surgeons, especially when there are comminuted articular fragments [3, 4].

Although efficacy is related to many factors, the surgical approach is one of the most important intervention factors for complex distal tibial fractures [2, 3, 5, 6]. For ease of fracture reduction, a variety of surgical incisions have been employed in the treatment of various ankle fractures [5–9]. Typically, severe ankle fractures (AO/OTA type 44) and distal tibial plafond fractures (AO/OTA type 43) are caused by combined axial load and valgus or rotational force. Sometimes, they are characterized by concomitant fibular fracture and distal tibiofibular syndesmosis injury. In such fractures, a conventional single incision is usually insufficient for direct visualization of the articular fragments, especially the whole articular surface, while a more visible combined incision carries an increased risk of narrow skin bridge necrosis [5, 10].

Inspired by ankle arthrodesis via the transfibular approach [11], we hypothesized that utilizing the broken lateral structure injured in the original injury could provide ideal visualization of the articular fragments, especially the articular surface, to facilitate the reduction of some complex fractures. Then, we employed a novel approach that provided direct visualization of the fibular notch to treat specific ankle fractures. The purpose of the present study was to describe the approach in detail and to present a series of patients with clinical and radiographic outcomes.

## Materials and methods

### Patients

The present study was approved by the Institutional Review Board of our hospital, and informed consent was obtained from all participants. Between March 2015 and October 2018, we treated 167 ankle fractures involving the distal tibial plafond at 1 regional level-1 trauma center. We retrospectively analyzed the data of all patients. Finally, 22 patients were identified and included in this study. The inclusion criteria were as follows: comminuted distal tibial plafond fracture with concomitant fibula and distal tibiofibular syndesmosis injury, definitive open reduction internal fixation (ORIF) performed using a fibular notch approach as the primary approach, adult fracture, and a minimum of 12 months of follow-up. The exclusion criteria included open fractures with a wound around the ipsilateral distal fibula and ORIF performed using another approach as the primary approach. Of all 22 patients, 16 were male, and 6 were female. The mean age was 42.9 ± 10.6 years (range 22 to 60). The mean follow-up time was 18.7 ± 4.3 months (range 12 to 24). The relevant information is listed in Table 1. Injuries were caused by motor vehicle accidents (MVA 7), falling down from a high place (Fall 7), sprains on the stairs (Sprain 4), heavy pounding (Pound 3), and crush injuries (Crush 1). According to the AO/OTA classification, there were 5 patients with 43-B2 fractures, 4 with 43-B3 fractures, 3 with 43-C2 fractures, 6 with 44-C1 fractures, 3 with 44-C2 fractures, and 1 with 44-C3 fractures. Demographic data, mechanism of injury, fracture AO/OTA classification, complications (delayed wound healing, infection, bone nonunion, PTOA), and ankle range of motion (ROM) were reviewed from the inpatient and outpatient medical records. The details of the surgical technique were reviewed from the operative notes.

**Table 1:** Summation of the patient and outcome data

Age	Mechanism	Follow-up (month)	Classification	Preop interval (day)	Reduction quality		Complications	Bone union (week)	Ankle ROM	Lateral stability	AOFAS	VAS
					Fracture	Syndesmosis						
51	Fall	24	43 B3	6	MM	Normal	PTOA	20	20DF–35PF	Normal	80	1
40	MVA	17	43 B2	7	AR	Normal	–	16	20DF–35PF	Normal	100	0
22	Pound	24	44 C2	5	AR	Normal	PTOA	16	25DF–35PF	Normal	80	1
37	Fall	18	44 C1	0	AR	Normal	–	16	25DF–40PF	Normal	100	0
33	Sprain	20	44 C2	9	AR	Normal	–	16	25DF–35PF	Normal	100	0
56	Sprain	18	43 B3	55	AR	Normal	–	20	10DF–25PF	Normal	90	0
32	Crush	12	43 C2	7	MM	Normal	–	16	20DF–40PF	Abnormal	80	1
27	MVA	18	44 C1	0	AR	Normal	–	16	30DF–40PF	Normal	100	0
41	MVA	18	43 C2	11	AR	Normal	–	20	25DF–30PF	Normal	88	0
44	MVA	18	43 B2	7	AR	Normal	–	16	25DF–35PF	Normal	97	0
57	Pound	13	43 B3	0	AR	Normal	DWH, SI	24	15DF–25PF	Normal	76	3
60	Fall	24	43 C2	10	MM	Normal	PTOA	20	25DF–35PF	Normal	86	0
50	Pound	13	44 C1	0	AR	Normal	–	16	20DF–40PF	Normal	94	0
46	Sprain	20	44 C2	7	AR	Normal	–	16	25DF–45PF	Normal	100	0
45	Fall	12	43 B2	10	AR	Normal	–	16	20DF–40PF	Normal	84	0
53	MVA	24	44 C1	8	AR	Normal		28	10DF–25PF	Normal	78	2
29	Fall	18	43 B2	0	MM	Normal	PTOA	14	25DF–40PF	Normal	80	1
55	Sprain	24	44 C1	7	AR	Normal		16	20DF–25PF	Normal	84	1
39	Fall	15	44 C1	9	AR	Normal	–	16	25DF–35PF	Normal	100	0
42	MVA	23	43 B3	9	AR	Normal	–	16	25DF–40PF	Normal	82	2
54	MVA	20	44C3	10	MM	Normal	–	20	15DF–20PF	Normal	84	0
30	Fall	12	43 B2	7	AR	Normal	–	16	25DF–35PF	Normal	90	0

### Surgical technique

In patients with reliable soft tissue around the ankle, definitive osteosynthesis was performed in an emergency; otherwise, plaster, calcaneus traction or a temporary ankle-spanning external fixator was applied in patients with traumatized soft tissue. The normal length and rotation of the extremity were maintained, and definitive internal fixation was performed after a positive “wrinkle sign” arose.

Under anesthesia, after distal tibiofibular syndesmosis injury was finally confirmed by lateral rotational stress radiography, the patient was placed in a floating position with a tourniquet placed on the proximal thigh. A longitudinal incision was made at the posterior border of the fibula from the proximal fibular fracture line to the tip of the fibula. In patients with anterior tibial plafond compression, the skin incision was extended towards the base of the fourth metatarsal.

Anterior blunt extraperiosteal dissection over the fibula was performed to obtain the anterolateral interval. Through this interval, the fractured fibula, the torn anterior inferior tibiofibular ligament (AITFL) and interosseous membrane, the lateral column, and the Tillaux fragment of the tibia were observed. The distal fibula was stretched with a clamp, obvious instability was observed, and lateral rotation was attempted. The anterior talofibular ligament (ATFL) obstructing the displacement of the distal fibula was revealed and was cut off in most patients. Subsequently, the distal fibula hinging on the posterior structures, including the posterior inferior tibiofibular ligament (PITFL), the calcaneofibular ligament (CFL), and the posterior talofibular ligament (PTFL), was retracted posteriorly, similar to the open-book technique. Held by the assistant, as a joystick, a K-wire inserted into the distal fibula provided great convenience for open-book manipulation. Sometimes, a lamina spreader was employed to maintain the lateral rotational position of the fibula. Then, the fibular notch was completely exposed. With the talus in an adducted position and the contents of the anterior compartment elevated, the lateral column of the tibia together with the whole plafond articular surface, even the horizontal surface of the medial malleolus, were visualized directly. Usually, progressing from proximal to distal, the tibial metaphysis, the Tillaux fragment, the die-punch fragments, and the Volkmann fragment were reduced visually and fixed with multiple temporary K-wires, screws, or buttress plates. Autogenous iliac bone grafts were sometimes applied for disimpacted metaphysis to support the tiny articular fragments, and micro-screws (2.0 mm or 2.7 mm) were sometimes used for stabilization in some fractures. Using the talar dome and fibular notch as references, the reduced articular fragments and articular surface were examined under direct visualization. The fibular fracture and the syndesmosis were reduced and temporarily fixed with K-wires at this point, but this was not the final fixation because of the following procedure.

**Fig. 1:**
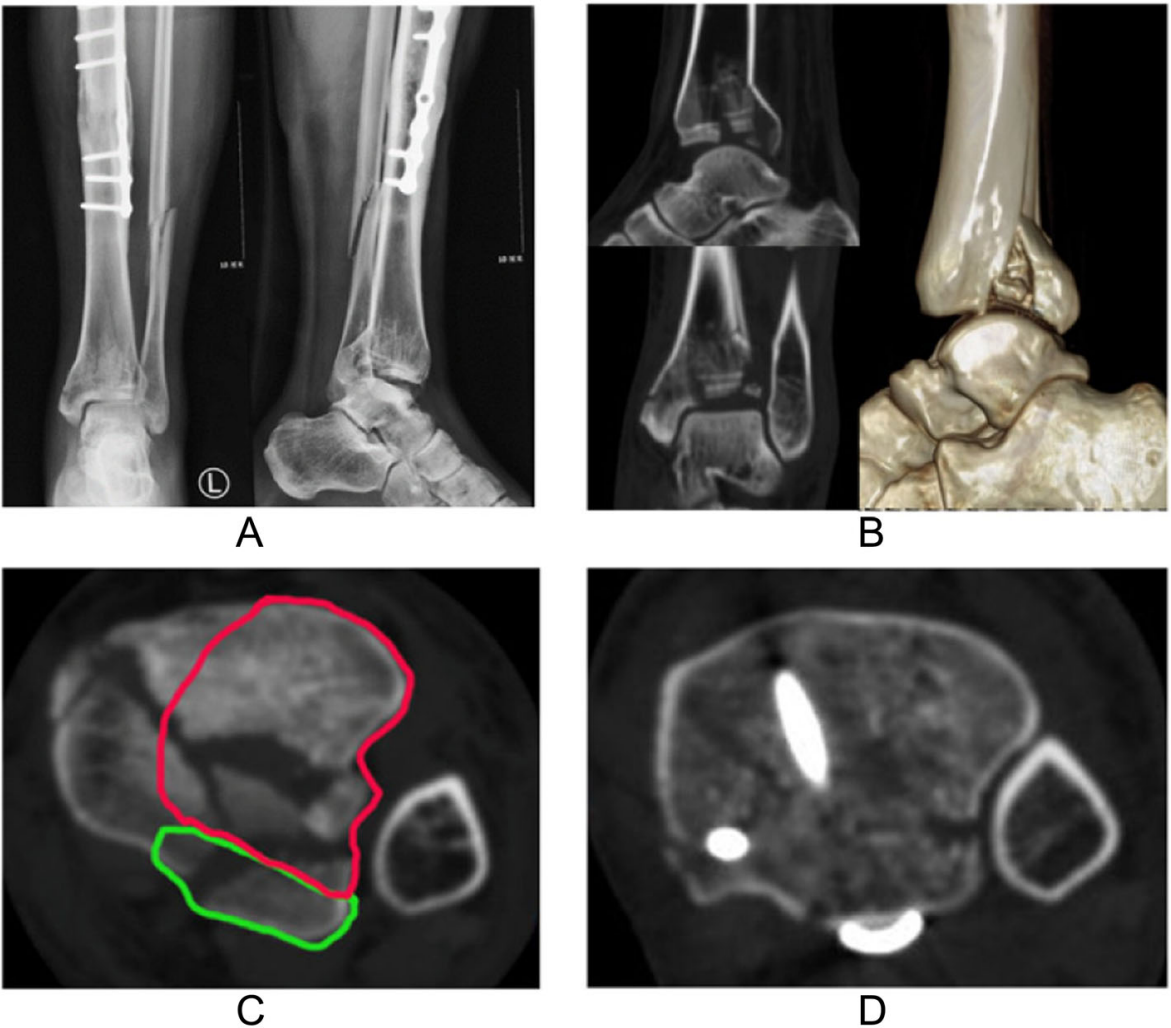
Imaging data of a 44-year-old male patient. **a** AP and lateral radiographs present AO/OTA 43 B2 fracture, with the ipsilateral tibia having previously undergone plate fixation. **b** Preoperative CT plain scan and reconstruction after removal of fibula. **c** Axial CT image 5 mm proximal to the tibial plafond shows comminuted fracture; red and green curve lines show areas that can be exposed intraoperatively through the anterior and posterior intervals, respectively. **d** CT image in the same position as **c** shows good reduction of the fragments and distal syndesmosis

**Fig. 2:**
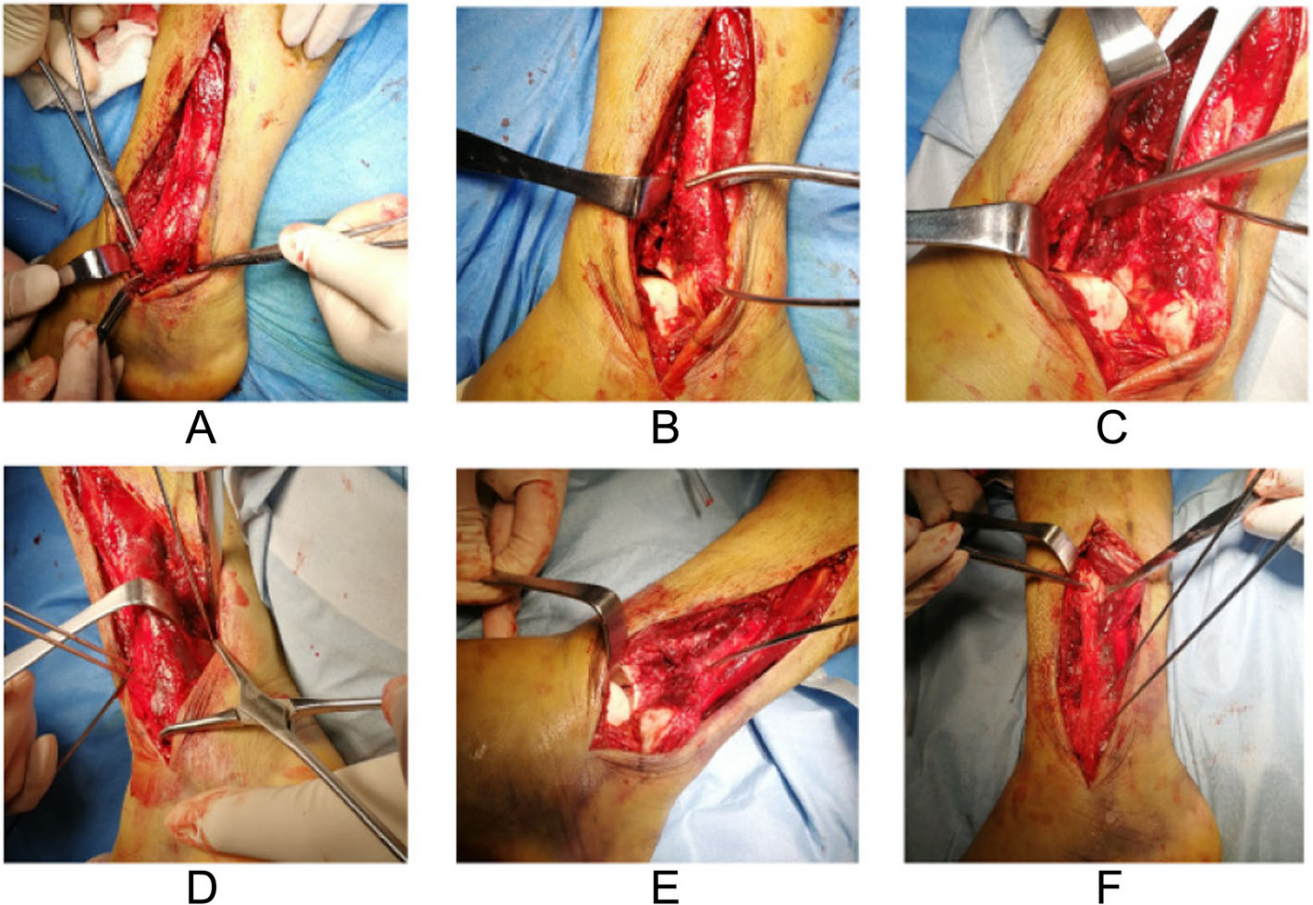
The surgical process of the same patient shown in Fig. 1. **a** Cutting the ATFL. **b** Posteriorly rotated fibula and good visualization of articular fragments. **c** Fracture reduction in anterior interval. **d** Temporarily fixed fibula and manipulation in posterior interval. **e** Second-look to articular surface. **f** Reduced fibula and syndesmosis

Next, between the flexor hallucis longus muscle and peroneus tendon, posterior blunt dissection was performed to obtain the posterior interval. The posterior interval was the same as the interval in a standard posterolateral (PL) approach, which is considered to provide optimal exposure of the posterior ankle [12]. Using the restored anterior components of the distal tibia as a template, the posterior column and the Volkmann fragment were further reduced and fixed with buttress plates or posteroanterior screws. Subsequently, the temporary K-wires fixing the syndesmosis were removed, and the fibula was turned laterally for the second time. Definitive restoration of the articular fragments and the articular surface was identified with a “second-look.” Then, alignment of the tibia and matching of the tibiofibular joint were confirmed by an interoperative image intensifier. Mechanically appropriate internal fixators were employed to complete the fixation of the metaphysis and lateral column of the tibia as well as the fibula. The distal tibiofibular syndesmosis was typically stabilized by one or two trans-syndesmotic screws across three cortices. The AITFL and the ATFL were carefully repaired by sutures or augmented by anchors. The incision was routinely drained and sutured. Alternately, if needed, minimally invasive osteosynthesis with a small medial incision or percutaneous screw osteosynthesis was applied for the treatment of a concomitant medial malleolar fracture. The ruptured medial deltoid ligament was carefully repaired (Figs. 1 and 2).

### Postoperative treatment

**Fig. 3:**
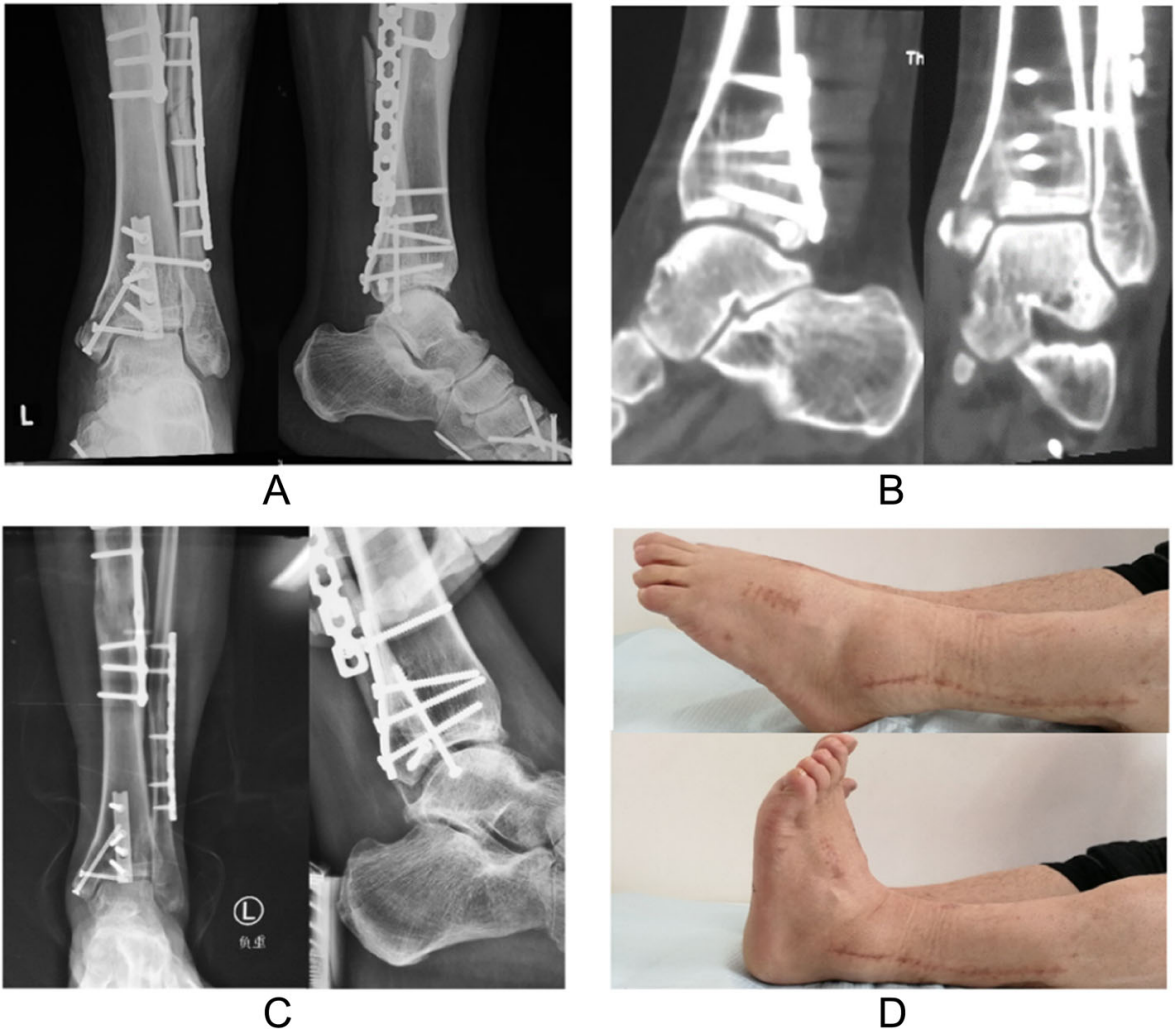
Postoperative data of the same patient shown in Fig. 1. **a** AP and lateral radiographs 1 week after surgery. **b** CT images 1 week after surgery. **c** Weight-bearing AP radiograph and stress lateral radiograph 6 months after surgery present normal ankle stability. **d** Ankle ROM 1 year after surgery

Postoperatively, the ankle was immobilized in a neutral position using a below-the-knee splint. The splint was removed 3 weeks after surgery, and active range-of-motion exercises were advocated. Postoperative radiography and CT scans were performed within 1 week after surgery. The reduction quality of the fracture and syndesmosis was examined using CT scans. For the fracture, anatomical reduction (AR) was considered in the case of a fracture gap or step-off of less than 2 mm; 2–5 mm was considered mild malreduction (MM), and more than 5 mm was considered severe malreduction. For syndesmosis, the same standard as Gardner’s [13] criteria, a greater than 2-mm difference on the CT scan between the anterior and posterior tibiofibular distances was considered malreduction. Ten to 12 weeks postoperatively, the second round of radiography was performed, and the syndesmosis screws were removed under local anesthesia. Fracture union was considered to be achieved by bridging callus maturation and closure of more than three quarters of the fracture faces on radiographs and the absence of pain during full weight-bearing [14]. One year after surgery or at the last follow-up, lateral stability of the ankle, including distal syndesmosis and the ATFL, was assessed by careful manual stress examination. In suspected patients, weight-bearing AP radiograph for syndesmosis and anterior drawer test stress lateral radiograph for the ATFL were performed. Absent tibiofibular overlap and a greater than 3 mm difference in the distance between the center of the plafond and the nearest point of the talus on lateral radiographic comparison of the uninjured and injured ankles were considered to be diagnostic criteria for lateral instability of the ankle [15]. Ankle ROA was measured with a goniometer, and the American Orthopedic Foot and Ankle Society Ankle-Hindfoot Scale (AOFAS) score was implemented for clinical functional evaluation (Fig. 3).

## Results

Four patients with reliable soft tissue underwent definitive osteosynthesis in an emergency, and the others had an interval from injury to definitive ORIF of 8.1 ± 1.7 days (range 5 to 11), except for a patients with an old fracture who had not been treated for 55 days after injury. Postoperative radiography revealed satisfactory restoration of all fractures; however, postoperative CT showed mild malreduction (MM) with a gap of less than 2 mm in 4 fractures and a step-off of less than 2 mm in 1 fracture. No patient had severe fracture malreduction or abnormal syndesmosis reduction. Delayed wound healing (DWH) and superficial infection (SI) were revealed in one patient who had an injury due to heavy pounding, and there were no cases of deep infection. Complete bone union was achieved in all cases with an average time of 17.3 ± 3.6 weeks (range 14 to 28). Mild PTOA was present in 4 patients during the relatively short follow-up period in this study, and all patients had an injury mechanism of falling down. The average ankle ROM was 21.6 ± 5.2° (range 10 to 30) in dorsiflexion and 33.9 ± 6.2° (range 20 to 40) in plantarflexion, and the average total motion arc was 55.0 ± 11.3° (range 35 to 70). All patients achieved lateral stability of the ankle except one patient who had painful syndesmosis instability with absent tibiofibular overlap on weight-bearing radiographs. The symptoms disappeared when his distal syndesmosis was stabilized by the suture button 1 year after surgery. The average AOFAS score and VAS score of all patients was 88.8 ± 8.6 (range 76 to 100) and 0.55 ± 0.86(range 0 to 3) at the last follow-up, see Table 1.

## Discussion

Our main objective was to observe the convenience of the fibular notch approach in the treatment of complex ankle fractures and its effect on fracture reduction and efficacy. It should be noted that the cases in the current study included both classical pilon fractures (AO/OTA 43-B and 43-C) and special ankle fractures (AO/OTA 44-C), although strictly speaking, it might not be appropriate to discuss the fractures as a whole. Sometimes, ankle fractures involving the distal tibial plafond are caused by a combined injury mechanism, which typically leads to a confusing classification of the fracture, such as a “logspatter” injury [16] versus an atypical pilon fracture, as well as a posterior malleolus fracture versus a “posterior pilon” fracture [17]. We would like to use the words “fractures involving the distal tibial plafond” rather than “tibia pilon fractures” to describe the confusing ankle fractures in the present study.

There are two points of concern in the treatment of these complex fractures. One is to try to reconstruct the anatomic bony structure, including the articular surface and metaphysis, to prevent traumatic arthritis, and the other is to make a safe incision on the traumatized and thin surrounding soft tissue to avoid soft tissue complications [9, 18]. However, a single incision bears the defect of inadequate exposure of the joint, while double incisions not in the diagonal axis of the ankle sometimes fail to meet the requirement of a distance of 7 cm between incisions [18–20]. A variety of approaches, including anterolateral [21], posterolateral [6], and lateral approaches [12], have been well described. We acknowledge that most fractures in the distal tibia can be treated through traditional approaches; however, these approaches do not seem to be a solution for all fractures, especially when a severely comminuted intra-articular fracture is present [7]. Because of the obstruction of the distal fibula and the talus, it is actually difficult to obtain sufficient operative visualization of the die-punch fragments through a traditional single incision, while multiple approaches for direct ORIF may result in a high risk of soft tissue complications [22]. As a result, with the absence of intraoperative CT to help examine reduction quality, poor restoration of the articular surface is not uncommon, even in patients treated by skilled surgeons [12].

Since Adams JC [11] described the transfibular approach for ankle arthrodesis in 1948, the transfibular approach has been widely used by Napiontek M [23] and Akra GA et al [24], with the best advantage of fully exposing the articular surface of the ankle. Inspired by this approach, we tried to apply a novel approach similar to it, the fibular notch approach introduced in the present study, for the treatment of some specific fractures. An epidemiological study showed that syndesmotic injury can occur in up to 20% of classical ankle fractures [25] and 15% of pilon fractures [26]. This provides surgeons an opportunity to utilize the injured lateral structure to fully expose the most difficult but critical articular surface without significantly increasing iatrogenic injury. Evaluation of the fibular incisura of the tibia with magnetic resonance imaging shows two thirds of the fibular notch is blocked by the lateral malleolus [27], and it is the exact main factor blocking the exposure of the articular surface in the widely used anterolateral (AL) and posterolateral (PL) approaches.

Anatomically, the distal tibia and fibula are tightly linked by four syndesmotic ligaments, and the distal fibula is further enhanced by the lateral ankle ligament complex [28]. While using the fibular notch approach, the ATFL is the only possible ligament that sometimes needs to be cut off for sufficient exposure of the fracture. The other ligaments, including the PITFL, the CFL, and the PTFL, which extend obliquely from anterior-lateral to posterior-medial, are very unlikely to be involved in the original injury (less than 10% involved) [29, 30] and will not be obstacles to the intraoperative lateral rotation of the distal fibula. Typically, the AL approach is widely used in fractures involving the anterolateral components of the distal tibia with good visualization, and the PL approach is more advantageous for the treatment of posterior column and Volkmann fragments. The fibular notch approach is a combination of the two and has both advantages. As the fibula is rotated posteriorly, an anterior interval similar to but broader than that in the AL approach can be obtained. With the first temporary reduction of the fibula, the same posterior interval as is achieved in the traditional PL approach can be achieved. Of particular significance, it is convenient to retract the distal fibula again to open the syndesmosis to examine the articular fragments and the articular surface in suspicious patients without blocked visualization due to the reduced and temporarily fixed fragments at the articular margins [8]. The posterior-anterior-posterior process of retracting the distal fibula and the “second look” technique have the advantage of avoiding the embarrassment caused by uneven articular surfaces on postoperative CT scans. It should be noted that the two intervals in the fibular notch approach are not equally effective. The posterior interval is essentially designed to assist the anterior interval for facilitating fixation from the posterior to the anterior direction. Therefore, although the incision is located at the posterior border of the fibula rather than at the traditional midway point between the Achilles tendon and lateral malleolus [12], it is not difficult to complete manipulation behind the ankle.

To our knowledge, four similar incisions or approaches have been described in prior articles. The first is the transfibular approach used for ankle arthrodesis [31]. It is also a real approach that completely exposes the fibular notch, and the distal fibula is completely or partially amputated rather than temporarily removed. The second is the trans-fibular-fracture approach described by Gonzalez TA for fixation of ankle fractures [19]. In this approach, all manipulations of distal tibial exploration and reduction are performed through the fracture line of the fibula. It is not suitable for low fibula tip fractures (Weber A ankle fractures) or high-level transverse fibular fractures, and the fibular notch is not exposed. The third is an oblique incision that was introduced by Niall P et al [32] in 2016 for simultaneous open reduction and internal fixation of the posterior malleolus and anterior syndesmosis. The authors fixed posterior malleolar fractures via the window between the peroneal tendons and the posterior aspect of the fibula and explored anterior syndesmosis via dissection anterior to the fibula. The authors also did not mention the exposure of the fibular notch. The last is a case report published in January 2019 during the follow-up of our case [33]. The authors used almost the same approach as ours to treat a tibial pilon fracture where only the lateral part of the distal tibia was affected. The incision was made along the anterior border of the fibula, and the distal fibula was retracted posteriorly to explore the lateral aspect of the tibial pilon. Unlike us, the author did not make a posterior interval to address the posterior ankle fracture, nor did they show the posterior-anterior-posterior shift of the distal fibula or utilize the “second look” technique, while these manipulations are of paramount importance for the observation of complex intra-articular fractures. In addition, the authors did not describe the ATFL closely related to the retracted fibula.

Most patients achieved good functional recovery, which benefited from the anatomical reduction of the tibial articular fragments, inferior tibiofibular syndesmosis, and lateral malleolus. With direct visualization of the fibular notch approach, shortening and rotation of the fibula and occult mismatch of the inferior tibiofibular syndesmosis can be avoided. However, four patients with falling injuries still had mild PTOA, which might be related to the original injury of the articular cartilage, and this relatively short follow-up time still needs to be further extended to increase reliability. Overall, a low rate of soft tissue complications was presented in our study, except for one patient with crushed soft tissue who had delayed wound healing and superficial infection, which supported the idea that a single incision had a lower risk of soft tissue complications.

We acknowledge that the fibular notch approach has some drawbacks. It has fewer indications and is not a conventional approach. It can only be considered when comminuted tibial plafond fractures are combined with fibular fractures and distal tibiofibular syndesmosis injuries, that is, specific Weber type C fractures caused by axial load and rotational force are more likely to be associated with the indications. Additionally, except in a few patients in which the ATFL was broken in the original injury, the ATFL was cut off, which may potentially have increased the tendency for postoperative ankle instability. Interestingly, with the soft tissue being repaired carefully, 4 patients in the present study complained of slight ankle stiffness and no one complained of ankle instability. Perhaps it is the same as cutting off the CFL in an extensive lateral approach to a calcaneal fracture, which is not prone to postoperative instability due to soft tissue contracture and scarring.

## Conclusions

The fibular notch approach is a safe and reliable approach for the treatment of specific ankle fractures involving the distal tibial plafond. This approach provides excellent direct visualization of the fragments and articular surface without significantly increasing iatrogenic injuries. Satisfactory radiographic and clinical results were observed, and further clinical and anatomical studies are recommended to ascertain the feasibility of this approach in the treatment of complex distal tibial fractures.

## Data Availability

All data used and analyzed during this study are available from the corresponding author on reasonable request.

## Abbreviations

AOFAS: American Orthopaedic Foot and Ankle Society
PTOA: Posttraumatic osteoarthritis
ORIF: Open reduction internal fixation
ROM: Range of motion
AITFL: Anterior inferior tibiofibular ligament
ATFL: Anterior talofibular ligament
PITFL: Posterior inferior tibiofibular ligament
CFL: Calcaneofibular ligament
PTFL: Posterior talofibular ligament
AL: Anterolateral
PL: Posterolateral
AR: Anatomical reduction
MM: Mild malreduction
MVA: Motor vehicle accidents
DWH: Delayed wound healing
SI: Superficial infection
